# Right Axillary Artery Cannulation for Veno-Arterial Extracorporeal Membrane Oxygenation in Postcardiotomy Patients: A Single-Center Experience

**DOI:** 10.3390/medicina59112040

**Published:** 2023-11-20

**Authors:** Medhat Radwan, Karim Baghdadi, Aron Frederik Popov, Rodrigo Sandoval Boburg, Petar Risteski, Christian Schlensak, Thomas Walter, Rafal Berger, Fabian Emrich

**Affiliations:** 1 Department of Thoracic and Cardiovascular Surgery, University Hospital of Tuebingen, 72076 Tuebingen, Germany; medhat.radwan@med.uni-tuebingen.de (M.R.); rodrigo.sandoval-boburg@med.uni-tuebingen.de (R.S.B.); christian.schlensak@med.uni-tuebingen.de (C.S.); 2 Department of Thoracic and Cardiovascular Surgery, Johann-Wolfgang-Goethe University, 60590 Frankfurt am Main, Germanythomas.walter@kgu.de (T.W.); fabian.emrich@kgu.de (F.E.); 3 Division for Cardiothoracic, Transplantation and Vascular Surgery, Hannover Medical School, 30625 Hannover, Germany; aronf.popov@gmail.com; 4 Department of Cardiac Surgery, University Hospital Zuerich, 8091 Zuerich, Switzerland; petar.risteski@usz.ch

**Keywords:** extracorporeal membrane oxygenation, ECMO, ECLS, heart failure, mechanical circulatory support

## Abstract

*Background and Objectives*: To analyze the patient outcome and complication rate of axillary artery cannulation for veno-arterial extracorporeal membrane oxygenation (VA-ECMO) in patients who could not be weaned from cardiopulmonary bypass after cardiothoracic surgery. *Materials and Methods:* We analyzed the data of 179 patients who were supported with VA-ECMO with femoral–axillary access (FA VA-ECMO) after cardiothoracic surgery between January 2014 and January 2019 in our department. Patients requiring central aortic cannulation and patients with respiratory failure requiring veno-venous ECMO were excluded. Primary outcomes were in-hospital mortality and 1-year survival rate of patients who were weaned from VA-ECMO support. Secondary outcomes were cannulation-related complications at the axillary site, VA-ECMO-related complications, and systemic complications. *Results:* In our cohort, 60 (33.5%) patients were female. Mean age was 67.0 ± 10.9 years. Overall, 78 (43.5%) patients were operated upon electively, 37 (20.7%) patients underwent urgent surgery, and 64 (35.8%) patients underwent emergency surgical treatment. Sixty-seven patients (37.4%) were resuscitated preoperatively. The mean duration of VA-ECMO support was 8.4 ± 5.1 days. Weaning from VA-ECMO was successful in 87 (48.6%) patients; 62 (34.6%) patients survived the hospital stay. The 1-year survival rate was 74%. Subclavian bleeding occurred in 24 (13.4%) patients, femoral bleeding in 4 (2.2%) patients, ischemia of the upper limb in 11 (6.1%) patients, intracerebral bleeding in 9 (5%) patients, and stroke in 19 (10.6%) patients. *Conclusions:* In patients with acute LV dysfunction after cardiothoracic surgery who cannot be weaned from cardiopulmonary bypass, right axillary artery cannulation is a safe and reliable method for VA-ECMO support with an acceptable complication rate.

## 1. Introduction

Postcardiotomy cardiogenic shock (PCCS) is defined as an inability to be weaned from cardiopulmonary bypass (CPB) during a cardiac surgery despite maximal inotropic support [1]. Prolonged cardiopulmonary support in these situations provides time for myocardial recovery that may reduce mortality from 100% without mechanical circulatory support (MCS). Veno-arterial extracorporeal membrane oxygenation (VA-ECMO) support through an axillary access provides circulatory support with antegrade flow while avoiding the need for keeping the chest open after cardiothoracic surgery [2].

Retrograde blood flow in the aorta during VA-ECMO with a femoral access may lead to insufficient left ventricular (LV) unloading in patients with severely impaired LV function [3]. Insufficient unloading can result in elevated LV end-diastolic pressure (LVEDP), pulmonary venous hypertension (PH), and may even lead to cardiogenic pulmonary edema followed by a watershed phenomenon with further supply of the heart with poorly oxygenated blood [4]. Furthermore, severely impaired LV contractility is associated with intra-cardiac stasis and can result in intra-cardiac thrombus formation [4]. In our center, the axillary cannulation for VA-ECMO is our standard access for ECLS support in PCCS patients, except in those where the closure of the chest was not possible due to bleeding complications or myocardial edema. The benefits of axillary artery cannulation include central support with antegrade flow and minimized risk cerebral embolization [5].

The aim of the study was to evaluate the in-hospital mortality and 1-year postoperative survival rate in patients who were treated with VA-ECMO with the axillary cannulation due to PCCS. Secondary objectives were to assess the complications that may have been caused by FA-VA-ECMO.

## 2. Materials and Methods

### 2.1. Patient Selection

We retrospectively analyzed the data of 179 patients who had been supported with VA-ECMO after cardiac surgery between January 2014 and January 2019 in the department of cardiothoracic surgery in the University of Frankfurt am Main hospital in Germany.

**Figure 1:**
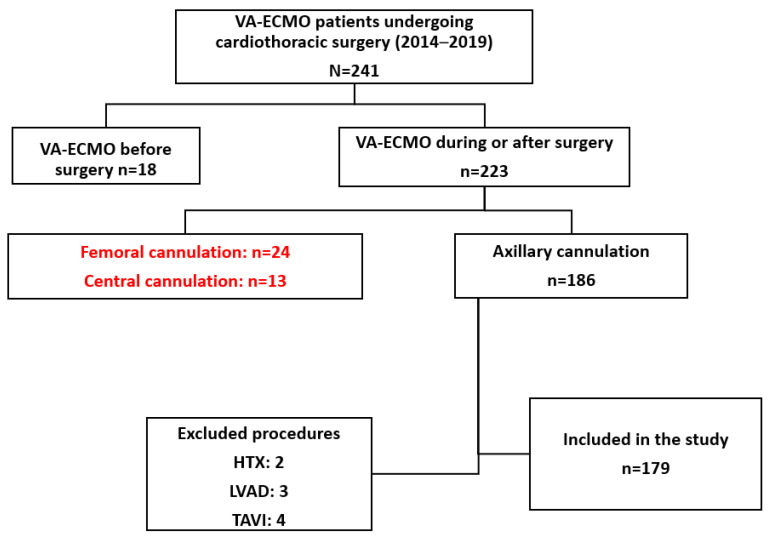
Patient selection process.

We excluded patients with PCCS requiring central aortic cannulation for the ECMO and patients with respiratory failure requiring veno-venous ECMO after cardiothoracic surgery. The patient selection process is shown in Figure 1.

### 2.2. ECMO Implantation

The ECMO therapy was initiated during the surgery if it was not possible to wean the patients from CPB despite maximal medical therapy and the patient showed signs of PCCS. The indication followed an interdisciplinary decision between the surgical and anesthesiologic team. The venous cannula was implanted percutaneously through the femoral vein. We did not use a vascular graft sewn with an “end-to-side” technique. For the implantation of the arterial cannula, an incision was made under the clavicle, and the axillary artery was exposed and clamped proximally and distally. The axillary artery was then opened, and an arterial cannula (elongated one-piece arterial (EOPA, Medtronic, Brooklyn Park, MN, USA) was advanced (about 7 cm) into the arterial lumen and secured with polyester thread.

A 4- or 5-French sheath was inserted, distal to the cannulation site using the Seldinger technique, to allow a distal perfusion of the right upper extremity. The above-mentioned reperfusion sheath and the arterial cannula were connected through a dedicated line and then the cannulas were connected to the ECMO circuit and a gradual weaning from CPB was performed as the ECMO support began. The arterial cannula was fixated via non-absorbable sutures to the subcutaneous tissue. The skin was closed with interrupted sutures.

### 2.3. Management and Weaning Protocol

A systemic anticoagulation with unfractionated heparin (UFH) was performed and monitored daily with target aPTT of 50–70 s. VA-ECMO management and weaning were performed according to the criteria proposed by the Extracorporeal Life Support Organization guidelines [6].

### 2.4. VA-ECMO Complications

We defined ECMO-related complications in three categories:

1. Cannulation-related complications, including local bleeding, infection, and limb ischemia at the arterial cannulation site.
2. VA-ECMO-related complications, including systemic hemorrhagic complications, VA-ECMO membrane thrombosis, and pulmonary edema.
3. Systemic complications: pulmonary infection and sepsis.

Axillary bleeding was defined as a clinical local swelling and high drainage volume requiring surgical revision. Limb ischemia was defined as a pale and cold limb due to decreased limb perfusion, requiring surgical revision with cannula relocation or removal. Cannulation site infection was defined as local signs of sepsis and positive culture of the axillary access requiring surgical revision. Systemic hemorrhagic complications were defined as any gastrointestinal bleeding.

### 2.5. Statistical Analysis

Data were analyzed using SPSS 26.0, (IBM Corporation, Armonk, NY, USA). Parameters are presented as whole values. Variables were tested for normality using the Kolmogorov–Smirnov test and Shapiro–Wilk test. Values with a normal distribution are represented as mean and standard deviation; values which do not represent a normal distribution are represented as mean (Q1–Q3).

### 2.6. International Review Board

The international review board/ethics committee of Frankfurt approved this project with the number 513/15 on 15 June 2022. Due to the retrospective nature of this study written consent was not necessary.

## 3. Results

Baseline demographic and cardiac surgical procedures performed are listed in Table 1.

**Figure 2:**
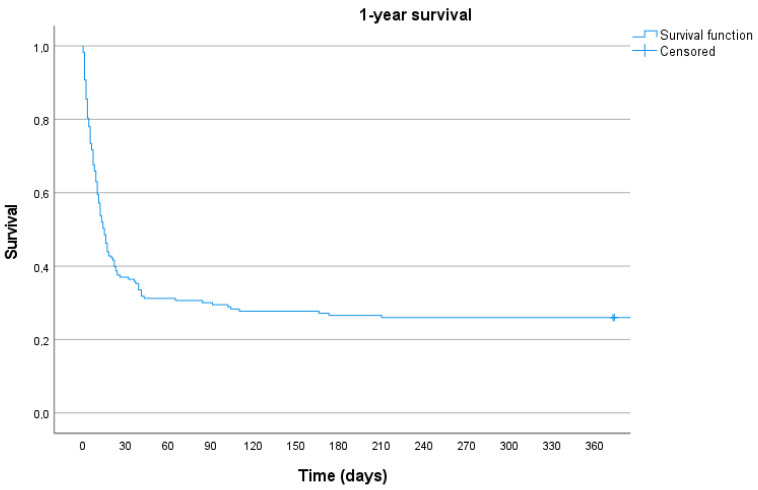
Kaplan–Meier curve depicting the 1-year survival of patients with FA VA-ECMO after PCCS.

The mean duration of VA-ECMO support was 8.4 ± 5.1 days. Mean intensive care unit stay was 18.8 ± 15.8 days and mean hospital stay was 23.48 ± 22.6. Weaning from VA-ECMO was successful in 87 (48.6%) patients, hospital survival was achieved in 62 (34.6%) patients, and 1-year survival was observed in 46 (25.7%) patients. Results are shown in Table 2 and in Figure 2.

Subclavian bleeding occurred in 24 (13.4%) patients, femoral bleeding in 4 (2.2%), re-sternotomy due to bleeding in 20 (11.1%), ischemia of the upper limb in 11 (6.1%), lower limb in 13 (7.2%), renal replacement therapy was needed postoperatively in 150 (83.8%), intracerebral bleeding occurred in 9 (5%), and stroke in 19 (10.6%). These results are shown in Table 3.

## 4. Discussion

PCCS occurs in up to 6% of patients after a cardiac surgery [7]. A prolonged MCS applied after a cardiac surgery to treat a PCCS helps reduce the periprocedural mortality providing a survival benefit [8,9]. VA-ECMO therapy due to cardiogenic shock (CS) has shown satisfying long-term results in non-cardiac surgical patients [10]. If MCS is needed at the intensive care unit (ICU), a percutaneous femoro-femoral access is the most reliable and convenient method of implantation without a need for a transfer into an operating theater; it can be performed at the patient’s bed without direct involvement of cardiac or vascular surgeons. However, femoral arterial access is associated with a broad spectrum of complications such as limb ischemia, perforation or dissection of the artery, and the risk of differential hypoxia (Harlequin’s syndrome) [8].

If CS ensues during or directly after cardiac surgery, there are other possibilities for achieving VA-ECMO support, for example via the axillary or subclavian artery [11]. In patients supported by VA-ECMO, the partial pressure of oxygen in the radial artery is similar to that achieved in central aortic cannulation, resulting in better upper body oxygenation compared to femoral arterial perfusion [12]. Another alternative to postoperative mechanical support is the possibility of direct cannulation of the ascending aorta. In that setting, there is a need to re-open the chest for decannulation or to leave a prosthetic material in the chest, which bears additional complications. Unlike percutaneous femoral cannulation, axillary arterial access can be technically challenging but provides a central blood flow with reduced risk of cerebral embolization [5].

We report a successful weaning in 48.6% of patients and an in-hospital survival of 34.6%; these results compare to those reported in the literature by other groups [13]. Our data show that nearly 50% of the patients requiring VA-ECMO after cardiac surgery could be weaned, despite suffering preoperative cardiac decompensation, due to myocardial recovery. Forty-six (25.8%) patients were alive after 1-year follow-up.

Bleeding requiring surgical interventions was the most common complication and occurred in 11.17% of patients, and was lower than what other groups have reported for femoral arterial cannulation of up to 22% [14]. Transient upper limb ischemia occurred in 6.7% of cases and an upper limb compartment was observed in 3.35% of patients. When comparing these numbers to those reported for femoral cannulation, it is evident that the rate of vascular complications is much lower than that has been reported so far, with rates ranging from 10 to 70% [12].

A therapeutic anticoagulation with UFH was performed on a routine basis with aPTT monitored daily. There are only a few studies proposing alternative anticoagulant medication [15]. It was not the objective of our study to evaluate the effectiveness of anticoagulation, but complications such as bleeding or thrombosis may result from insufficient or overdosed UFH [16].

We did not observe hyper-flow syndrome and there was no need for temporary banding of peripheral axillary artery and no need for a limb amputation in any case of VA-ECMO access in our cohort. An axillary cannulation is especially useful for patients at risk for limb ischemia, especially those with peripheral vascular disease, diabetes, or with signs of peripheral malperfusion before initiation of VA-ECMO therapy [13].

Another possible and important complication of peripheral VA-ECMO support is a disparate perfusion of the lower and upper parts of the body, also known as the watershed phenomenon or Harlequin’s syndrome [17]. In the study of Radakovic et al. comparing peripheral and central cannulation in 158 consecutive patients, the rate of disparate perfusion was 14.8% in the group with peripheral femoral perfusion, followed by a necessary intervention, and 0% in the group with central arterial cannulation [17]. We did not observe any case of disparate body perfusion in our series of axillary cannulation either; in any case, there was no need for further changes in the perfusion technique.

The infection of the cannulation site is another common problem of VA-ECMO support. With a mean therapy time of 8.4 ± 5.1 days, a local infection was observed in 4.47% of cases. Infectious groin complications are common after femoral arterial access and are observed in 20% of survivors, resulting in prolonged length of hospitalization [18].

We decided to remove the patients with central cannulation from further analysis. In this specific sub-group of patients, the course of VA-ECMO therapy will be different and the expected complications, such as mediastinitis due to the implantation of prosthetic grafts and bleeding, require the thoracotomy to be re-attempted. Another sub-population was the patients who underwent heart transplant while on VA-ECMO support. The survival of this cohort differs from the survival of non-transplanted individuals due to complications specific to immunosuppressive therapy, and the timing of transplant surgery depends on the time of organ availability being an independent factor with irrelevant duration of MCS.

### Limitations

Our study is limited to a single-center retrospective experience. We included only patients after a cardiac surgical procedure with PCCS as a complication of the initial procedure. The study cohort was not homogeneous and included both elective and urgent patients who underwent any kind of cardiac surgery. The population size was limited and there is no control group comparison. However, due to the limitations we could avoid a multi-center bias regarding various strategies for cannulation during VA-ECMO support.

## 5. Conclusions

Our data suggest that subclavian artery cannulation provides safe and perhaps improved access for providing VA-ECMO support and facilitating myocardial recovery in patients requiring MCS after cardiac surgery.

## Figures and Tables

**Table 1:** Demographical data.

	N	Percentage
Sex		
Male	119	66.48%
Female	60	33.52%
Age (y)	67.05 ± 10.9	
BMI (kg/m^2^)	33.18 ± 48.61	
Indication		
CABG	72	40.22%
Valvular Pathology	52	29.05%
Combined CABG and Valvular Pathology	28	15.64%
Thoracic Aorta	20	11.17%
Other	7	3.91%
Chronic Atrial Fibrillation	33	17.74%
Hypertension	121	67.60%
Diabetes	79	44.13%
Pulmonary Hypertension	85	47.49%
Dyslipidemia	43	24.02%
LVEF (%)		
>50	74	41.34%
50–31	48	26.82%
30–21	23	12.85%
<20	22	12.29%
NYHA III-IV	137	76.54%
Acute MI	54	30.17%
CPR	69	38.55%
Intubated Admission	33	18.44%
Type of Surgery		
Elective	74	41.34%
Urgent	37	20.67%
Emergency	64	35.75%
CPB time (min)	196.08	
Cross Clamp Time (min)	101.50	

**Table 2:** Mechanical circulatory support characteristics.

	Number	Percentage
ECMO Duration (d)	8.37 ± 5.13	
Implantation during CPR	32	17.88%
Successful ECMO weaning	87	48.60%
Hospital stay (d)	23.48 ±22.66	
ICU stay (d)	18.80 ± 15.86	
Discharged	62	36.31%
Mortality during ECMO	92	51.40%
Mortality after ECMO	44	24.58%

CPR: cardiopulmonary resuscitation; ECMO: extracorporeal membrane oxygenation; ICU: intensive care unit.

**Table 3:** ECLS-related complications.

	Number	Percentage
Access site bleeding	20	11.1%
Cerebral bleeding	7	3.91%
Ischemia	29	16.20%
Upper limb	12	6.70%
Lower limb	13	7.26%
Both	4	2.23%
Compartment syndrome	11	6.15%
Upper limb	6	3.35%
Lower limb	4	2.23%
Both	1	0.56%
Cannulation site infection	5	4.47%
Stroke	11	6.15%

## Data Availability

Data are available on request.
